# Intermittent High Glucose Enhances Apoptosis in INS-1 Cells

**DOI:** 10.1155/2011/754673

**Published:** 2011-06-21

**Authors:** Xiao-li Shi, Yue-zhong Ren, Jing Wu

**Affiliations:** ^1^Department of Endocrinology, Second Affiliated Hospital of Medical College, Zhejiang University, Hangzhou 310009, China; ^2^Department of Endocrinology, Hangzhou Binjiang Hospital, Hangzhou 310009, China

## Abstract

To investigate the effect of intermittent high glucose (IHG) and sustained high glucose (SHG) on inducing **β**-cell apoptosis and the potential involved mechanisms, INS-1 beta cells were incubated for 72 h in the medium containing different glucose concentrations: control (5.5 mmol/L), SHG (33.3 mmol/L), and IHG (5.5 mmol/L and 33.3 mmol/L glucose alternating every 12 h). Cell viability, apoptosis rate, and oxidative-stress markers were determined. The results showed that the apoptosis induced by IHG was more obvious than that by SHG. Simultaneously, the intracellular level of oxidative stress was more significantly increased in INS-1 cells exposed to IHG. These findings suggest that intermittent high glucose could be more deleterious to **β**-cell than a constant high concentration of glucose, this may be due to the aggravation of oxidative stress triggered by intermittent high glucose.

## 1. Introduction

Apoptosis, an active process of cell suicide morphologically and biochemically different from necrosis and actively regulated, plays an important role in the development of diabetes. In recent years, the apoptosis of *β*-cells has become a major focus. Several lines of evidence from autopsy suggested that *β*-cell mass in diabetic patients is significantly reduced and its reduction is associated with increased apoptosis [1–3]. Both animal and human studies have found that increase of *β*-cell apoptosis is an important reason of insulin deficiency in patients with T2DM [1, 4]. So far, numerous evidences have suggested that chronic persistent hyperglycemia results in *β*-cells dysfunction and ultimately apoptosis, called *β*-cell glucose toxicity [5]. However, the previous researches mostly focused on the toxic effect of persistent high glucose, in fact, in addition to chronic persistent hyperglycemia, another general and important phenomenon is the repeated fluctuation in blood glucose that existed in diabetes patients which is more excessive than in healthy individuals [6]. Recently, a few published reports demonstrated the effects of intermittent high glucose on the development of diabetic complications [7, 8], but the effect of intermittent high glucose on *β*-cell and the potential mechanisms involved remain unknown.

There are many potential mechanisms whereby excess glucose metabolites traveling along these pathways might cause *β*-cell damage. However, all these pathways have in common the formation of reactive oxygen species that cause chronic oxidative stress, which in turn causes increased apoptosis [9]. However, few reports show whether intermittent high glucose triggers the oxidative stress within pancreatic *β*-cell.

In the present study, we therefore aimed at analyzing the role of intermittent high glucose in inducing *β*-cell apoptosis and exploring the implications of the chronic oxidative stress in this process.

## 2. Materials and Methods

### 2.1. Chemicals

RPMI1640 medium and fetal bovine serum were purchased from GIBCO. 2′, 7′-dichlorofluorescein diacetate (DCFH-DA), dimethyl sulphoxide (DMSO), 3-(4, 5-dimethylthialzal-z-yl)-2,5-diphenylterazolium bromide (MTT), and Hoechst 33258 dye were purchased from Sigma, Annexin V: FITC apoptosis detection kit was form BD. Malondialdehyde (MDA) assay kit and glutathione (GSH) assay kit were products of the Institute of Nanjing Jiancheng Biology Engineering (Nanjing, China).

### 2.2. *β*-Cell Culture

Dr. Tong-feng Zhao kindly supplied INS-1 cells. The cells were cultured in RPMI-1640 medium with 11 mmol/L D-glucose supplemented with 10% fetal bovine serum (FBS), 100 U/mL penicillin, 100 *μ*g/mL streptomycin, 10 mmol/L HEPES, 2 mmol/L L-glutamine, 1 mmol/L sodium pyruvate, and 50 *μ*mol/L 2-mercaptoethanol. After being cultured overnight in glucose-free RPMI 1640 with 5.5 mmol/L glucose, INS-1 cells were divided into three groups: (1) control group (CG) exposed to normal concentration of glucose (5.5 mmol/L), (2) stable high glucose (SHG) group exposed to 33.3 mmol/L glucose, and (3) intermittent high glucose (IHG) group exposed to fluctuating concentrations of glucose (alternating between 5.5 mmol/L and 33.3 mmol/L glucose every 12 h), and were incubated for 72 h.

### 2.3. Cell Viability Assay

Cell viability was estimated by measuring the metabolism of 3-(4, 5-dimethyldiazol-2-yl)-2, 5-diphenyltetrazolium bromide (MTT). INS-1 cells were seeded in 96-well microtiter plates with 1 × 10^3^ per well and incubated for 72 h. At the end of the exposure, 20 *μ*L of MTT solution (5 mg/mL) was added to each well, and cells were maintained for 4 h at 37°C. After the supernatant fluid was removed, 150 *μ*L of solubilization solution (DMSO) was added to each well and shaken for 10 min. Absorbance was measured at 570 nm, with a microplate reader. Cell viability was expressed as a percentage of cytoprotection, versus control group set at 100%.

### 2.4. Morphological Analysis by Hoechst 33258

To further address the death pattern, INS-1 cells were stained with a nuclear dye Hoechst 33258. The Hoechst 33258 fluorochrome is sensitive to chromatin and is used to assess the changes in nuclear morphology. After exposure for 72 h, INS-1 cells were stained with Hoechst 33258 (5 *μ*g/mL) for 30 min at room temperature in the dark. After washing three times with phosphate-buffered saline, cells were visualized and photographed under ultraviolet illumination using fluorescent microscopy. Nuclear morphological changes characteristic of apoptosis such as condensed or fragmented nuclei with strong bright Hoechst 33258 dye staining were observed. Ten random frames were imaged using fluorescence microscope for each group, and the number of total cells and apoptotic cells were obtained by counting. The percentage of apoptotic cells was calculated as follows:

(1) $$\text{\%}\;\text{apoptotic}\;\text{cells}\;=\;\frac{\text{the}\;\text{number}\;\text{of}\;\text{apoptotic}\;\text{cells}}{\text{the}\;\text{number}\;\text{of}\;\text{all}\;\text{cells}}.$$

### 2.5. Detection of Apoptotic Cells with Flow Cytometry

Apoptosis was assessed by annexin V-FITC and PI staining followed by analysis with flow cytometry. The methodology followed the procedures as described in the kit. Briefly, wash cells twice with cold PBS. Resuspend cells in 1 × binding buffer at a concentration of 1 × 10^6^ cells/mL and transfer 100 *μ*L (1 × 10^5^ cells) to a 5 mL culture tube. Then add 5 *μ*L of annexin V-FITC and 10 *μ*L of PI. Gently vortex the tube and incubate for 15 minutes at room temperature in the dark. Later, 400 *μ*L of 1 × binding buffer was added to each tube. The stained cells were analyzed by flow cytometry as soon as possible (within an hour).

### 2.6. Measurement of Reactive Oxygen Species (ROS) Production

The generation of ROS was measured using an oxidation-sensitive fluorescent probe 2′, 7′-dichlorodihydrofluorescin diacetate (DCFH-DA). Intracellular esterase activity results in the formation of DCFH, a nonfluorescent compound, which emits fluorescence when it is oxidized to DCF. DCFH-DA has been widely used to measure the formation of reactive species in cells [10]. Cells were incubated with 20 *μ*M of DCFH-DA for 30 min at 37°C, and then visualized under a fluorescence microscope or analyzed by flow cytometry. Mean fluorescence intensity was obtained.

### 2.7. Measurement of Malondialdehyde (MDA) and Glutathione (GSH)

The concentration of MDA was used as an index of lipid peroxidation. Cells were lysed by an ultrasonic cell disruptor and centrifuged at 10000 g at 4°C for 10 min. MDA was determined as thiobarbituric acid reactive substances (TBARS). In the assay, MDA reacts with thiobarbituric acid (TBA), yielding a stable chromophoric product with maximal absorbance at 532 nm. Results were expressed as nmol MDA per mg of protein. Protein concentration was determined by the BCA method (Beyotime, China).

 Glutathione (GSH) concentration in the cells was measured using the glutathione assay kit according to the instruction manual. The method is based on the development of a stable yellow color when 2-nitrobenzoic acid is added to sulfhydryl compounds. The amount of reduced product, thionitrobenzene, was measured at 412 nm and expressed as mg/gprot.

### 2.8. Statistical Analysis

All data were expressed as mean ± SD. Differences among experimental groups were evaluated by one-way analysis of variance (ANOVA), and the post hoc test (Fisher's PLSD) was used for multiple comparisons. Significance was defined as *P* < .05.

## 3. Results

### 3.1. IHG Decreased the Cell Viability in INS-1 Cells

**Figure 1:**
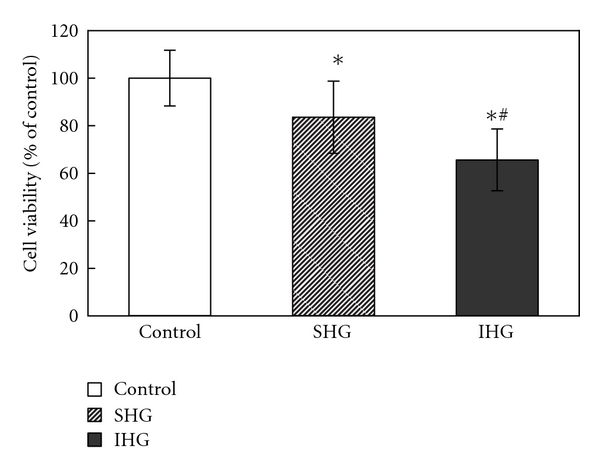
The cell viability in INS-1 cells. Cells were exposed to normal glucose, stable high glucose, and intermittent high glucose for 72 h. The results were presented as a percentage of control group viability. Data were expressed as mean ± SD. *P < .05 versus control, ^∗#^ *P* < .05 versus SHG.

The effect of IHG on cell viability in INS-1 cells was examined using the MTT reduction assay. As shown in Figure 1, the viability of INS-1 cells exposed to SHG and IHG was significantly reduced compared with control treatment (*P* < .05). Furthermore, IHG induced more significant decreases in cell viability than SHG (*P* < .05).

### 3.2. Morphological Assessment of Apoptosis by Hoechst 33258 Staining

**Figure 2:**
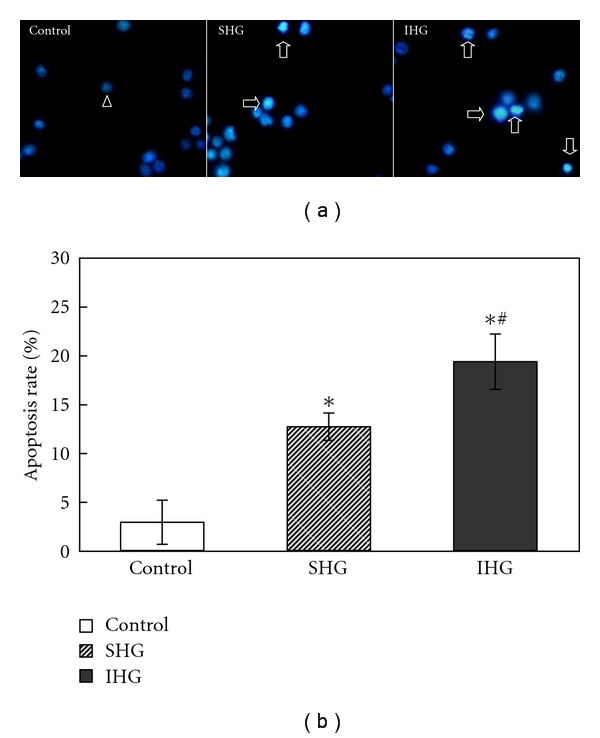
(a) Morphological assessment of apoptosis by Hoechst 33258 staining. Fluorescence microscope (400x) images. The arrowhead indicates normal nuclei and blank arrows indicate apoptotic nuclei. (b) Assessment of apoptosis by Hoechst 33258 staining. Apoptosis rate of each group was calculated as described in Section 2. The apoptosis rate was significantly increased in IHG group. Data were expressed as mean ± SD. *P < .05 versus control, ^∗#^ *P* < .05 versus SHG.

As shown in Figure 2(a), in normal cells, the nuclei exhibited dispersed and weak fluorescence, the apoptotic cells showed an apoptotic-like abnormal morphology (condensation and fragmentation) of nuclei and asymmetric staining with Hoechst 33258. As illustrated in Figure 2(b), after incubation for 72 h, about 2.89 ± 2.26% of INS-1 cells showed a typical cell apoptosis as revealed by typical chromatin condensation in control group. Cells incubated with SHG and IHG revealed the marked condensed chromatin, and the number was significantly increased compared with that of control group (*P* < .05), with apoptosis rate 12.67 ± 1.41% and 19.33 ± 2.83%, respectively. These above results indicated that the apoptotic rate in IHG group is higher than SHG group.

### 3.3. Induction of Apoptosis in INS-1 Cells Measured by Annexin-V/PI Staining via Flow Cytometry

**Figure 3:**
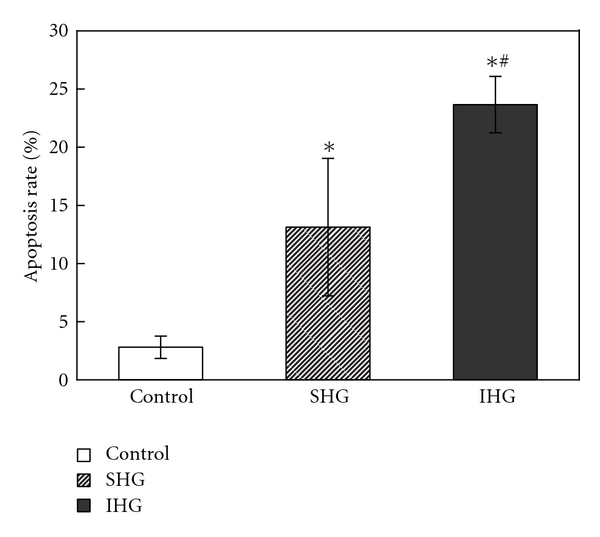
Detection of apoptotic cells with flow cytometry. After the treatment for 72 h, cells were collected for the determination of cell apoptosis as described previously. The percentage of apoptotic cells was shown after analysis by flow cytometry. Data were expressed as mean ± SD of three determinations. *P < .05 versus control, ^∗#^ *P* < .05 versus SHG.

The INS-1 cells apoptosis was confirmed by flow cytometry. As shown in Figure 3, the percentage of apoptotic cells was (2.79 ± 0.96)% in control group while SHG and IHG increased the apoptosis rate to (13.12 ± 5.91)% and (23.66 ± 2.42)%, respectively. IHG group showed statistical differences (*P* < .05) compared with the SHG group. That means intermittent high glucose produced a significant increase of cell apoptosis compared with both normal and stable high glucose concentrations.

### 3.4. IHG Increased Reactive Oxygen Species Level

**Figure 4:**
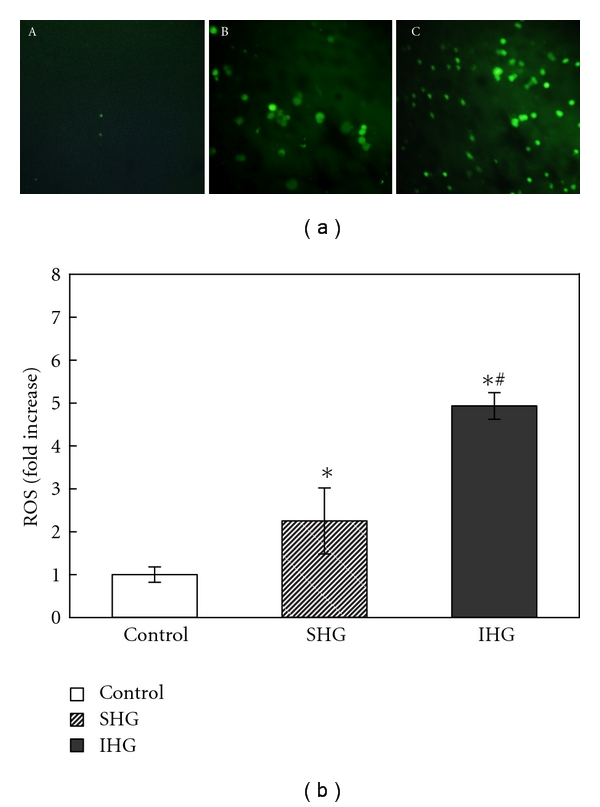
Demonstration of intracellular ROS of INS-1 cells with DCFH-DA staining. (a). Images of DCFH-DA staining for INS-1 cells with different treatment. The graph (A) control group, (B) INS-1 cells exposed to stable high glucose, and (C) INS-1 cells exposed to intermittent high glucose. (b). Data of DCFH-DA fluorescence intensity analysis, *P < .05 versus control, ^∗#^ *P* < .05 versus SHG.

To evaluate the influence of IHG on intercellular level of oxidative stress, intracellular reactive oxygen species level was measured by DCFH-DA fluorescence staining. As shown in Figure 4, when INS-1 cells were exposed to SHG, the intracellular reactive oxygen species level increased by 2.25-fold, more outstandingly, the reactive oxygen species level significantly increased by 4.93-fold when exposed to IHG.

### 3.5. IHG Increased the MDA Concentration and Decreased GSH Concentration in INS-1 Cells

As shown in Table 1, SHG and IHG treatment significantly increased MDA concentration, and the concentration was higher in IHG group compared to SHG group. The difference between two groups was significant (*P* < .05). Compared to control treatment, GSH concentration was decreased when exposed to SHG and IHG, but no significant differences were found between two groups.

## 4. Discussion

In the present study, we found that cultured INS-1 cells exposed to high glucose concentrations showed increased cell apoptosis, and this effect was further enhanced in cells that were exposed to intermittent rather than sustained high glucose. Furthermore, under the condition of IHG, the cellular oxidative stress was more significantly hyperactivated than with SHG treatment. These findings suggest that variability in glycemic control could be more deleterious to the INS-1 cells than a constant high glucose and oxidative stress may play an important role in mediating this process.

It is now clear that hyperglycemia not only causes *β*-cell dysfunction, but also induces *β*-cells apoptosis. Incubation of human pancreatic islets in the presence of high glucose concentrations and animal models with hyperglycemia has repeatedly documented an increase of apoptotic rate [10–15]. In the present study, evidence for apoptosis has been obtained by several methods. All methods confirm that high glucose concentrations lead to increased *β*-cell apoptosis. The result is in agreement with previous researches. More importantly, we emphasized the effect of fluctuating hyperglycemia. Although in normal individuals plasma glucose concentration is controlled strictly within a narrow range, in patients with prediabetes or diabetes, the plasma glucose concentration often changes markedly during the period of a single day [6, 16, 17]. At present, evidences demonstrated that intermittent high glucose may be more harmful than stable high glucose in the development of diabetic cardiovascular complications [18, 19]. Fluctuations of glucose display a more dangerous effect than stable high glucose on both tubulointerstitial and mesangial cells, in terms of cell proliferation [20, 21]. Similarly, compared to stable high glucose, intermittent high glucose more significantly enhanced apoptosis in human umbilical vein endothelial cells [22]. Consistently, our data showed that an intermittent exposure to hyperglycemia led to more effective induction of apoptosis in INS-1 cells than a persistent exposure. Thus cumulating evidence suggests that glucose variations may be more dangerous for the cells than stable high glucose. Recently, there are some evidences focused on the toxic effects of fluctuating high glucose on islet beta cell functions and survival [23, 24]. Del Guerra and his companions obtained human pancreatic islets from nondiabetic and nonobese multiorgan donors, and cultured them for 5 days in continuous normal glucose concentration (5.5 mmol/L) or normal and high (16.7 mmol/L) glucose levels (alternating every 24 h). They found that intermittent high glucose caused a significant decrease of glucose-stimulated insulin secretion and apoptosis was also significantly increased by intermittent high glucose exposure [24]. In agreement with Del Guerra et al.'s research, the present study also shows a deleterious effect of fluctuating glucose for INS-1 cells. Further comparing with stable high glucose, intermittent high glucose induced higher apoptotic rates. Thus, results of the present study and others indicate an important role for intermittent high glucose in the pathology of DM. 

Previous studies investigating the effects of glycemic variability on diabetic complications have explored the potential mechanisms, such as the role of PKC and NAD(P)H-oxidase, ICAM-1,VCAM-1, and E-selectin expression changes and monocyte behavior [22, 25, 26]. Oxidative stress was considered to be one important mechanism involved. More recently, El-Osta et al. [27] found that transient exposure of aortic endothelial cells to hyperglycemia induced persistent epigenetic changes in the promoter of the NF-*κ*B p65 subunit. These epigenetic changes were caused by increased generation of methylglyoxal because of hyperglycemia-induced ROS formation. Toxic oxidative stress is also an important reason for *β*-cell apoptosis [28]. It is particularly relevant and dangerous for the islet, which is among those tissues that have the lowest levels of intrinsic antioxidant defenses. As the hyperglycemia of diabetes becomes chronic, the sugar takes on the darker role of toxin and causes chronic oxidative stress. The present study found that high glucose produced an increase in oxidative stress generation. Moreover, it appears noteworthy that intermittent high glucose enhances oxidative stress generation compared to stable high glucose. This is consistent with previous studies showing that in patients with impaired glucose tolerance (IGT) and diabetes, fluctuation of plasma glucose concentration during postprandial periods exhibited a more specific triggering effect on oxidative stress than sustained hyperglycemia [17, 29]. 

The oxidative stress being a major mechanism for glucose toxicity in beta cells has been suggested by previous studies. High glucose concentrations increase the mitochondrial proton gradient as a result of overproduction of electron donors by the tricarboxylic acid cycle, which in turn increase production of mitochondrial superoxide [9]. Several other pathways of glucose metabolism such as glyceraldehyde autoxidation pathway and hexosamine pathway are also emphasized as being major contributors of ROS. 

ROS can interact with nearby cellular components, such as proteins, lipidse and DNA and results in wide-ranging damage. Oxidative modification of susceptible unsaturated fatty acids can result in the generation of lipid peroxides, and malondialdehyde (MDA) is one of them. GSH is one of the primary defense systems against oxidative stress. So ROS, MDA, and GSH can serve as markers of oxidative stress. In this study, we selected them to evaluate the levels of oxidative stress. Our data confirmed that the potential mechanisms involved in the toxic effects of IHG on pancreatic *β*-cells are, at least in part, the same as those working in stable high glucose concentrations. However, they are enhanced in intermittent high glucose condition.

In summary, our study suggested for the first time that chronic exposure to IHG led to more effective induction of apoptosis in INS-1 cells than SHG, which may be due to excessive activation of cellular oxidative stress. In our opinion, these data support the hypothesis that glucose fluctuation may be involved in *β*-cell injury. Moreover, our data suggest that this phenomenon is partly related to the activation of oxidative stress.

## Figures and Tables

**Table 1:** Mean values of concentrations of MDA and GSH in INS-1 cells with different intervention.

Group	MDA (nmol/mgprot)	GSH (mg/gprot)
Control	2.24 ± 0.41	45.50 ± 8.17
SHG	3.23 ± 0.73*	26.30 ± 10.73*
IHG	4.38 ± 1.10^∗#^	25.51 ± 7.74*

*P < .05 versus control, ^∗#^ *P* < .05 versus SHG.
